# Expression and Prognostic Role of Glia Maturation Factor-γ in Gliomas

**DOI:** 10.3389/fnmol.2022.906762

**Published:** 2022-06-29

**Authors:** Junhui Liu, Xiaonan Zhu, Lun Gao, Rongxin Geng, Xiang Tao, Haitao Xu, Zhibiao Chen

**Affiliations:** ^1^Department of Neurosurgery, Renmin Hospital of Wuhan University, Wuhan, China; ^2^Central Laboratory, Renmin Hospital of Wuhan University, Wuhan, China

**Keywords:** GMFG, gliomas, prognosis, immune, temozolomide

## Abstract

**Background:**

Glia maturation factor-γ (GMFG) regulates actin cytoskeletal organization and promotes the invasion of cancer cells. However, its expression pattern and molecular function in gliomas have not been clearly defined.

**Methods:**

In this study, public datasets comprising 2,518 gliomas samples were used to explore GMFG expression and its correlation with malignancy in gliomas. Immunohistochemistry (IHC) staining was performed to determine the expression of GMFG in gliomas using an in-house cohort that contained 120 gliomas samples. Gene ontology enrichment analysis was conducted using the DAVID tool. The correlation between GMFG expression and immune cell infiltration was evaluated using TIMER, Tumor Immune Single-Cell Hub (TISCH) database, and IHC staining assays. The Kaplan–Meier analysis was performed to determine the prognostic role of GMFG and its association with temozolomide (TMZ) response in gliomas.

**Results:**

The GMFG expression was higher in gliomas compared with non-tumor brain tissues both in public datasets and in-house cohort. High expression of GMFG was significantly associated with WHO grade IV, IDH 1/2 wild-type, and mesenchymal (ME) subtypes. Bioinformatic prediction and IHC analysis revealed that GMFG expression obviously correlated with the macrophage marker CD163 in gliomas. Moreover, both lower grade glioma (LGG) and glioblastoma multiforme (GBM) patients with high GMFG expression had shorter overall survival than those with low GMFG expression. These results indicate that GMFG may be a therapeutic target for the treatment of such patients. Patients with low GMFG expression who received chemotherapy had a longer survival time than those with high GMFG expression. For patients who received ion radiotherapy (IR) only, the GMFG expression level had no effect on the overall survival neither in CGGA and TCGA datasets.

**Conclusion:**

The GMFG is a novel prognostic biomarker for patients with both LGG and GBM. Increased GMFG expression is associated with tumor-associated macrophages (TAMs) infiltration and with a bad response to TMZ treatment.

## Introduction

Glioblastoma multiforme (GBM) is the most common malignant primary brain tumor with high mortality rates. The prognosis of GBM WHO grade IV is poor and its incidence is the highest of all malignant brain tumors (Ostrom et al., 2020). For instance, the median survival time of patients with GBM is approximately 1 year, and the overall 5-year survival is less than 5% (Ostrom et al., 2018). Temozolomide (TMZ) is one of the most effective chemotherapeutic agents used for the treatment of GBM. However, data show that the average survival time of GBM patients following radiation and TMZ treatment is lower than 15 months (Wang et al., 2019; Zheng et al., 2021). Immunotherapy has become a new treatment option for gliomas. The development of checkpoint blockade immunotherapy has revolutionized the treatment of GBM. Immune checkpoints regulate immune response and other molecules expressed by immune cells or tumor cells (Hombach-Klonisch et al., 2018; Maghrouni et al., 2021). Even though immunotherapy has clinical benefits for patients with GBM, many GBM patients do not respond sufficiently to checkpoint blockade (Desbaillets and Hottinger, 2021; Yu and Quail, 2021). Therefore, it is important to investigate the immune microenvironment of gliomas and identify new molecular markers, to improve gliomas treatment.

Glia maturation factor-γ (GMFG) is a small protein with 17 kDa. Its gene sequence is highly conserved from yeast to mammals (Kaplan et al., 1991). GMFG regulates the re-organization of actin cytoskeleton, as well as dipeptides that drive invasion and migration of cancer cells (Zuo et al., 2014; Wang et al., 2017). Studies have revealed that GMFG is mainly expressed in inflammatory cells and regulates the chemotaxis of neutrophils and lymphocytes (Aerbajinai et al., 2011). Aerbajinai et al. (2011) reported that GMFG was associated with the migration and polarity of neutrophils and depletion of GMFG in dHL-60 significantly reduced the CXCL8-induced chemotaxis. Elsewhere, knock-down of GMFG decreased the formation of lamellipodia in dHL-60 cells exposed to CXCL8 (Aerbajinai et al., 2011). GMFG has also been shown to inhibit cellular inflammatory signaling resulting leading to the suppression of monocyte chemotaxis by regulating the recycling of effective B-integrin to plasma membrane (Aerbajinai et al., 2016). It also influences the infiltration of immune cells and immune checkpoints; hence, it may be a novel therapeutic target for cancer treatment (Yang et al., 2021). Lan et al. (2021) reported the expression of GMFG in glioma for the first time and found that high expression of GMFG was associated with worse overall survival in glioma. However, the results of the findings were only from TCGA, and there is no multi-database sample verification. Currently, its expression pattern, association with molecular pathology, and immune cell infiltration in gliomas are elusive.

In this study, we explored the expression of GMFG in gliomas and its relationship with gliomas malignancy using public datasets and an in-house cohort. We found that the expression of GMFG in gliomas was significantly increased and correlated with tumor malignancy. Patients with high expression of GMFG had a worse prognosis compared with those with low expression of GMFG in TCGA, CGGA, Rembrandt, and Gravendeel datasets. Moreover, GMFG expression significantly correlated with macrophage infiltration and might play a role in influencing the gliomas microenvironment. Importantly, this study demonstrates that GMFG is a crucial marker for TMZ response in gliomas.

## Materials and Methods

### Clinical Samples

Paraffin-embedded gliomas tissue microarray comprising 120 gliomas samples and 10 non-tumor brain tissues was analyzed. All samples were acquired from hospitalized patients between January 2017 and March 2020 in the Department of Neurosurgery of Renmin Hospital of Wuhan University. None of them received any chemotherapy or radiotherapy before surgery. All patients signed informed consent. This study was approved by the Institutional Ethics Committee of the Faculty of Medicine at Renmin Hospital of Wuhan University [approval number: 2012LKSZ (010) H].

### Public Data Acquisition and Preprocessing

The GEPIA (Tang et al., 2017) and Oncomine^1^ registries (Rhodes et al., 2004) were analyzed to determine the mRNA expression pattern of GMFG in pan-cancers. Besides, the genomic alterations of the GMFG gene in TCGA-GBM and TCGA-LGG were explored using the cBioPortal platform^2^ (Cerami et al., 2012). Finally, the expression of GMFG in gliomas was analyzed in eight public datasets. The datasets included TCGA-LGG, TCGA-GBM, TCGA-GBMLGG, CGGA mRNAseq and Gravendeel (also known as GSE16011), Rembrandt, Gill, and Murat datasets; all obtained from GlioVis^3^ (Bowman et al., 2017). GlioVis website (see text footnote 3) is an important platform for data visualization and analysis to explore brain tumors. Except for normalized gene expression data, there are also information on glioma molecular pathology and GBM subtypes.

### Gene Function Enrichment Analysis

The top 100 genes that were positively correlated with GMFG (Spearman *r* > 0.50, *P* < 0.01) were downloaded from the cBioPortal platform (see text footnote 2) based on TCGA-GBM. The functional interactions among the GMFG-correlated genes described previously were combined with the functional annotation groups described in DAVID and Metascape.^4^

### Immune Estimation

Data on the correlation between gene expression and tumor-infiltrating immune cells were obtained from the TIMER platform^5^ (Li et al., 2020). This tool allows the assessment of immune infiltration, including TIMER, EPIC, and CIBERSORT, among others. Xcell results were selected. Data on immune cell markers were obtained from a previous study (Andersen et al., 2021). The correlation between GMFG expression and immune cell markers was determined based on the expression profile of TCGA and CGGA datasets. Estimation of STromal and Immune cells in MAlignant Tumor tissues was performed using Expression data (ESTIMATE) to predict tumor purity according to the gene expression profile. ESTIMATE algorithm is based on a single sample Gene Set Enrichment Analysis (Yoshihara et al., 2013). ESTIMATE generates three scores, including stromal score, immune score, and estimate score. The TCGA-GBM-based stromal score and immune score were calculated and downloaded for further use. Moreover, the Tumor Immune Single-Cell Hub (TISCH) database was employed to analyze the correlations between GMFG expression and infiltrating immune cells. TISCH is a scRNA-seq database focusing on the tumor microenvironment (Sun et al., 2021). This database contains the detailed information about cell-type annotation at the single-cell level and can be used to analyze the tumor microenvironment.

### Immunohistochemical Staining

The paraffin-embedded tissue microarray was heated in an oven (60°C) for 90 min. The slides were placed in xylene (5 min/time, 3 times) and ethanol at different concentrations (100, 95, and 75%) for hydration treatment. They were washed 3 times with phosphate-buffered saline (PBS) and incubated with 3% H_2_O_2_ for 10 min at room temperature to eliminate endogenous peroxidase. Slides were then completely immersed in the antigen retrieval liquid at 95°C for 10 min and allowed to cool naturally. Slides were treated with Triton-PBS (100×) for 5 min and blocked with 1% bovine serum albumin (BSA) for 30 min. The primary antibodies were added to the slides and incubated at 4°C overnight. The next day, the slides were washed with PBS (10 min/time, 3 times) and then incubated with horseradish peroxidase (HRP) goat anti-rabbit/mouse IgG for 1 h. The 3,3′-diaminobenzidine (DAB) reagent was added dropwise to the slides, and the color reaction was stopped with tap water. The slides were stained with hematoxylin reagent repeatedly for 1 min. The color was separated with a 1% hydrochloric acid alcohol solution. Finally, the slides were covered with a neutral balsam and observed using an Olympus BX40 microscope (Tokyo, Japan). Images were acquired for each group.

### Immunohistochemistry Tests for Immune-Reactive Cells

The intensity and percentage of immune-reactive cells were evaluated. The results were scored as follows: 0 denoted no staining, 1 denoted weak staining, 2 denoted moderate staining, and 3 denoted strong staining. Staining of GMFG was scored by the percentage of positive cells (0, <10%; 1, 10–25%; 2, 26–50%; 3, 51–75%; and 4, >75%). The final immunoreactive score (FIS) was calculated as follows: staining intensity × percentage of positive cells. We defined FIS (0–4) as low expression and FIS (6–12) as high expression. The immunohistochemistry (IHC) staining results were independently analyzed by two individuals.

### Statistical Analysis

Data are expressed as means ± standard deviations (SD) or ± standard error of the mean (SEM). Significant differences between the two groups were determined using a Student’s *t*-test. Multiple groups were compared with one-way ANOVA. All data analyzed by ANOVA or *t*-test appeared to have a Gaussian distribution and, therefore, parametric tests were used. The Spearman correlation analysis was performed to evaluate the correlation between parameters. The high- and low-expression groups were categorized based on the gene expression level at a given optimal cutoff value. Differences in survival between groups were analyzed using the Kaplan–Meier survival analysis with a log-rank significance test. The GraphPad Prism version 8.0 software (GraphPad Inc., San Diego, CA, United States) was used to create graphs.

## Results

### Glia Maturation Factor-γ Expression in Gliomas

**FIGURE 1:**
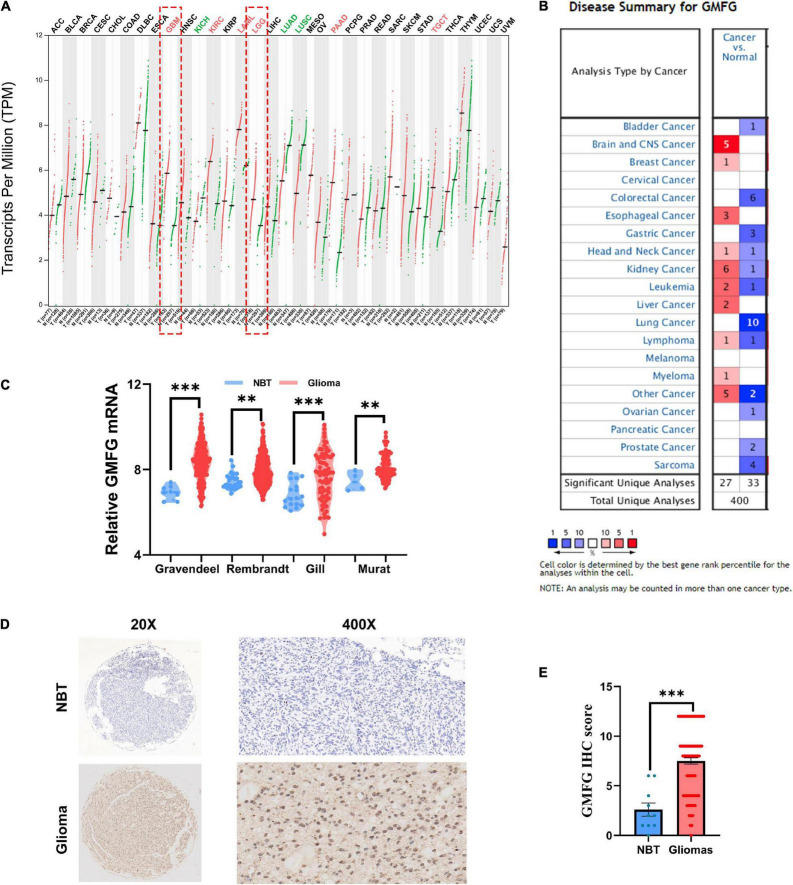
Glia maturation factor-γ (GMFG) expression in gliomas. **(A)** Transcripts of GMFG in pan-cancers in the GEPIA platform; **(B)** alterations to mRNA and protein levels of GMFG in different types of cancers in Oncomine; **(C)** relative mRNA level of GMFG in non-tumor brain tissues (NBT) and gliomas tissues from Gravendeel, Rembrandt, Gill, and Murat datasets; **(D,E)** immunohistochemistry (IHC) staining of GMFG in NBT and gliomas tissues. ^**^P < 0.01, ^***^P < 0.001, ns, no significance.

To compare the expression of GMFG between gliomas and normal brain tissues, the online databases GEPIA and Oncomine were used. Results of GEPIA showed that GMFG was significantly elevated in GBM and lower grade gliomas (LGG, WHO grades I–III) compared with normal brain tissues (Figure 1A). Analysis of GMFG expression in the Oncomine dataset revealed a significant increase in GMFG expression in brain and CNS cancer tissues (Figure 1B). To further explore the expression pattern of GMFG in gliomas, four public datasets, namely Gravendeel, Rembrandt, Gill, and Murat, were analyzed. Results showed that GMFG expression was higher in gliomas compared with normal brain tissues in the four datasets (Figure 1C). Furthermore, IHC analysis of the in-house cohort found that GMFG was mainly distributed in the nucleus and less so in the cytoplasm. The IHC data also indicated that GMFG expression was significantly higher in glioma tissues compared with non-tumor tissues (Figures 1D,E).

### Glia Maturation Factor-γ Expression Correlated With Malignancy of Gliomas

**FIGURE 2:**
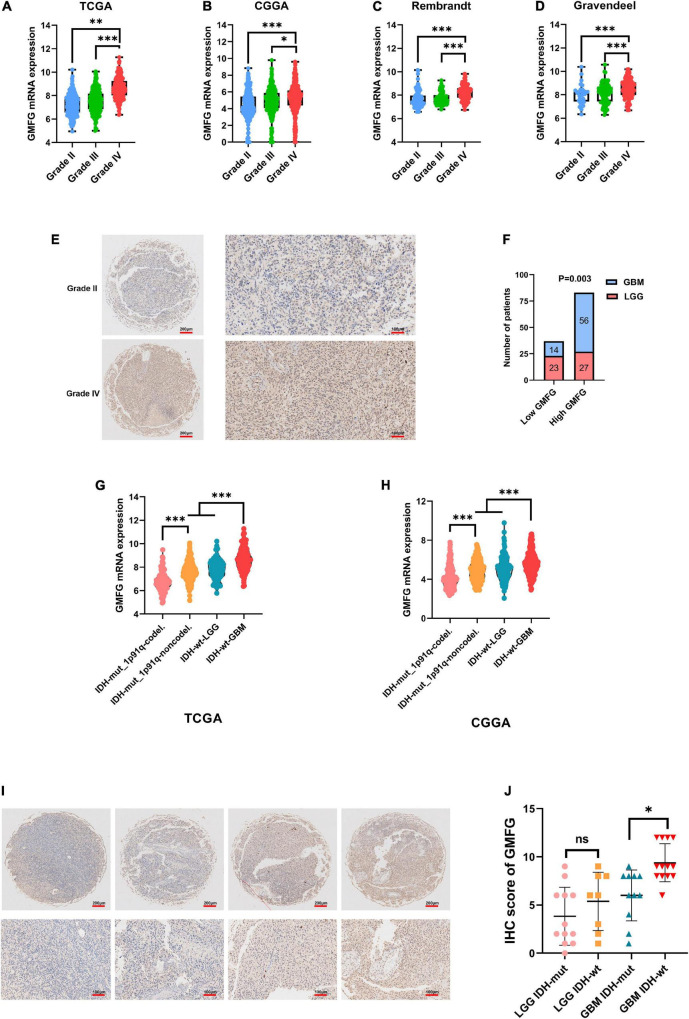
Glia maturation factor-γ expression correlated with malignancy of gliomas. **(A–D)** TCGA, CGGA, Gravendeel, and Rembrandt were used to investigate GMFG expression in gliomas. All data were downloaded from GlioVis platform. **(E,F)** IHC staining of GMFG in lower grade gliomas (LGG) and glioblastoma (GBM); **(G,H)** GMFG expression in gliomas with different IDH and 1p19q status in CGGA and TCGA datasets; **(I,J)** gene sequencing of glioma tissues from our validation cohort. GMFG expression in gliomas with different IDH and 1p19q status. mut, mutant; wt, wild-type. *P < 0.05, ^**^P < 0.01, ^***^P < 0.001, ns, no significance.

Gliomas are graded from WHO grade I to grade IV according to the degree of malignancy. Results showed that the GMFG expression increased with the grade in TCGA, CGGA, Gravendeel, and Rembrandt datasets (Figures 2A–D). In the in-house cohort, the IHC staining test revealed that GMFG expression was much higher in GBM tissues than in LGG tissues (Figures 2E,F). Two mutations (IDH1/2 mutations and 1p19q co-deletion) are routinely used for the diagnosis and classification of gliomas (Ludwig and Kornblum, 2017). Compared with those with IDH wild-type (IDH wt), gliomas with IDH-1/2 mutations have a favorable prognosis (Brandner and von Deimling, 2015). Compared with other molecular characteristics, GMFG expression was relatively higher in GBM with IDH wt both in TCGA and CGGA datasets (Figures 2G,H). Results of gene sequencing analysis showed that GMFG expression was significantly higher in IDH1/2 wild-type GBM than in LGG with or without IDH mutation (Figures 2I,J). The correlation between GMFG expression and clinicopathological characteristics of patients with gliomas in TCGA and in-house cohort is presented in Tables 1, 2, respectively.

**TABLE 1:** Correlation between GMFG and clinicopathological characteristics in patients with gliomas in TCGA.

Clinicopathological characteristics	GMFG expression		*P*-value
	Low (*n* = 321)	High (*n* = 348)	
Age	44.04 ± 13.29	50.24 ± 16.02	<0.001
Gender			
Female	129	125	>0.05
Male	158	197	
WHO grade			<0.001
I–III	276	194	
IV	15	135	
Subtypes			<0.001
ME	1	95	
Others	269	166	
IDH status			<0.001
Mutant	267	162	
Wild-type	52	181	
MGMT promoter			<0.001
Methylation	272	205	
Unmethylation	46	115	
TERT promoter			>0.05
Mutant	93	62	
Wild-type	99	67	
ATRX status			>0.05
Mutant	94	102	
Wild-type	225	238	
Chr.1p19q			<0.001
codeletion	143	26	
Non-codeletion	177	317	

**TABLE 2:** Correlation between GMFG and clinicopathological characteristics in patients with gliomas in in-house cohort.

Clinicopathological characteristics	GMFG expression		*P*-value
	Low (*n* = 36)	High (*n* = 84)	
Age	53.44 ± 9.78	55.26 ± 12.89	>0.05
Gender			
Female	19	38	>0.05
Male	17	46	
WHO grade			0.002
I–III	23	27	
IV	13	57	
IDH status			<0.05
Mutant	10	13	
Wild-type	3	18	
Chemotherapy			0.03
Yes	10	42	
No	26	42	
Radiotherapy			>0.05
Yes	7	21	
No	29	63	

### Glia Maturation Factor-γ Expression Associated With Glioblastoma Multiforme Subtypes

**FIGURE 3:**
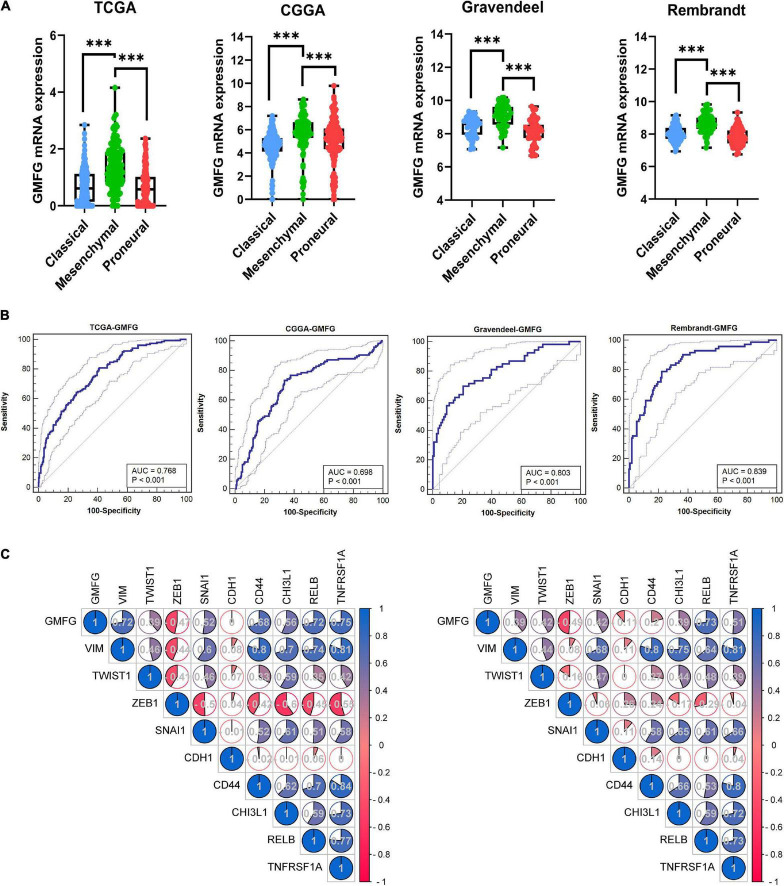
Glia maturation factor-γ expression associated with GBM subtypes. **(A)** GMFG expression in different subtypes of GBM in the TCGA, CGGA, Gravendeel, and Rembrandt datasets. CL, classical; ME, mesenchymal; PN, proneural. ^***^P < 0.001. **(B)** Accuracy of GMFG to predicting ME subtype as determined using ROC curves. ROC, receiver operating characteristic; AUC, area under the curve. **(C)** Spearman correlation method was employed to analyze the correlation coefficient between GMFG and mesenchymal-related genes in TCGA and CGGA.

Clinically, GBM is categorized into three subtypes [i.e., proneural, mesenchymal (ME), and classical subtypes] based on the molecular and phenotypic differences (Lee et al., 2018). The patients with ME GBM always correlated with relatively poor outcomes at diagnosis and at disease recurrence (Aldape et al., 2015). The expression of GMFG in TCGA, CGGA, Gravendeel, and Rembrandt molecular subtypes of GBM was explored. Results showed that GMFG expression was significantly higher in ME subtype GBM than in other subtypes (Figure 3A). The receiver operating characteristic (ROC) analysis was further performed to assess the prediction accuracy of GMFG for the ME subtype. Acceptable area under the curve (AUC) values of 0.768, 0.698, 0.803, and 0.839 were obtained for TCGA, CGGA, Gravendeel, and Rembrandt with respect to the prediction accuracy of GMFG expression for ME subtypes (Figure 3B). Next, the correlation between GMFG and ME-related genes was analyzed with the Spearman method. Results showed that GMFG expression positively correlated with vimentin, snail1, RELB, and TNFRSF1A, and negatively correlated with ZEB1 both in the TCGA and CGGA datasets (Figure 3C). Thus, we hypothesized that GMFG might regulate the transition from an epithelial to an ME phenotype.

### GO Functional Enrichment of Glia Maturation Factor-γ in Gliomas

**FIGURE 4:**
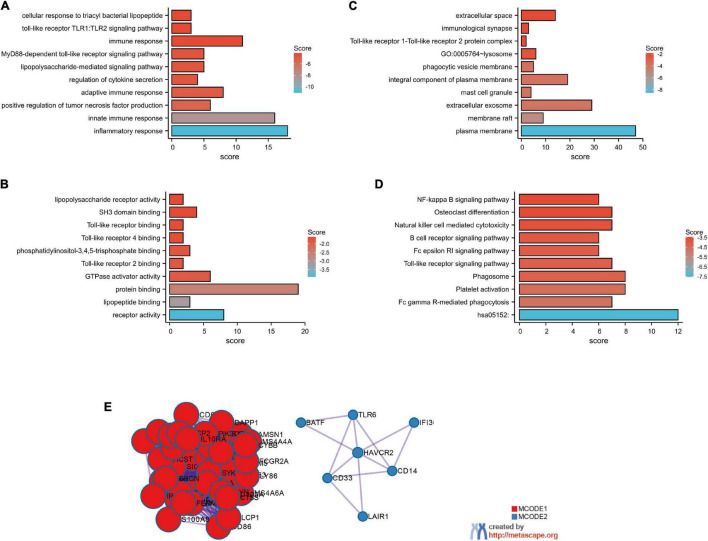
GO functional enrichment of GMFG-related terms in gliomas. The top 100 genes that were positively correlated with GMFG (Spearman *r* > 0.50, *P* < 0.01) were downloaded from the cBioPortal platform (http://www.cbioportal.org/) based on TCGA-GBM. GO enrichment analysis were classified into three. **(A)** Biological process; **(B)** molecular function; **(C)** cellular component categories. **(D)** KEGG pathways prediction. **(E)** Protein–protein interaction networks were performed in Metascape (http://metascape.org/).

The top 100 genes in the TCGA-GBM database with the strongest correlation with GMFG gene expression (Spearman’s correlation >0.50, *P* < 0.01) were determined using the cBioPortal online tool. Gene ontology enrichment was performed using DAVID to further analyze the function of GMFG. Results showed that the top-five enriched GO-biological process terms were as follows: GO:0006954 – inflammatory response, GO:0045087 – innate immune response, GO:0032760 – positive regulation of tumor necrosis factor production, GO:0002250 – adaptive immune response, and GO:0050707 – regulation of cytokine secretion (Figure 4A). For the molecular function GO component, the enriched terms were as follows: GO:0004872 – receptor activity, GO:0071723 – lipopeptide binding, GO:0005515 – protein binding, GO:0005096 – GTPase activator activity, and GO:0035663 – Toll-like receptor 2 binding (Figure 4B). For the cellular component of GO analysis, the enriched terms were as follows: GO:0005886 – plasma membrane, GO:0045121 – membrane raft, GO:0070062 – extracellular exosome, GO:0042629 – mast cell granule, and G GO:0005887 – integral component of plasma membrane (Figure 4C). In the KEGG pathway analysis, hsa05152:Tuberculosis, hsa04666:Fc gamma R-mediated phagocytosis, and hsa04611:Platelet activation were the enriched pathways (Figure 4D). A protein–protein interaction (PPI) analysis of GMFG-related genes described previously was performed in Metascape. Two significant gene modules were selected using the MCODE application (Figure 4E).

### Glia Maturation Factor-γ Correlated With Immune Cell Infiltration in Gliomas

**FIGURE 5:**
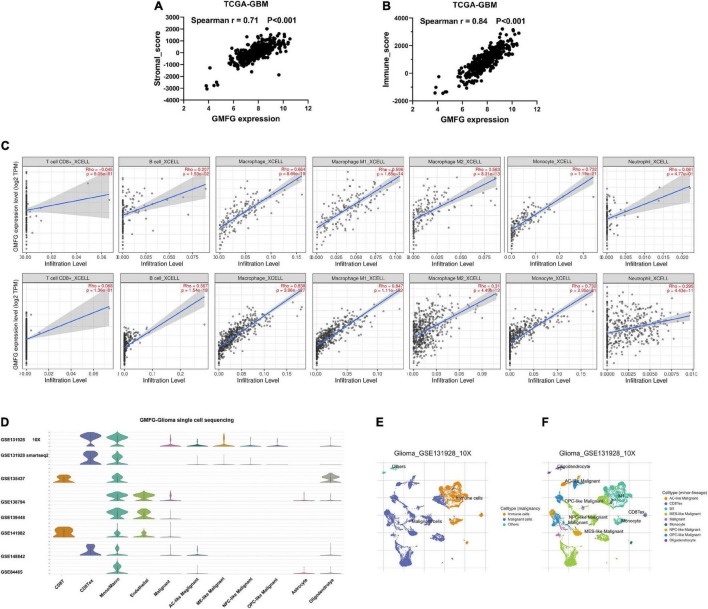
Glia maturation factor-γ correlated with immune cell infiltration in gliomas. Correlations between GMFG expression and stromal score **(A)** and immune score **(B)**. Stromal and immune scores were calculated by using the ESTIMATE. **(C)** Correlation between GMFG expression and the immune infiltration levels in TIMER. **(D–F)** Gliomas single-cell sequencing datasets from the TISCH database were used to explore the association between GMFG expression and immune cell infiltration.

Stromal and immune cells were recently identified to be fundamental components of the gliomas microenvironment, with a potential value for prognostic prediction and therapeutic application. Using ESTIMATE, we found that GMFG expression significantly correlated with a stromal and immune score in GBM (Spearman *r* = 0.71 and 0.84, respectively, Figures 5A,B). Meanwhile, the correlation between GMFG expression and the immune infiltration levels was evaluated in the TIMER. The level of GMFG expression correlated with high levels of immune infiltration of macrophage and monocyte both in LGG and GBM (Figure 5C). Furthermore, GMFG expression positively correlated with CD3D/CD3E (T-cell markers), CD86/CD79A/CSF1R (B cell), CCL2/CD68 (monocyte), CD163/IRF5/MS4A4A (macrophage), and ITGAM/CCR7 (neutrophil) both in the TCGA and CGGA databases. Moreover, T-cell exhaustion marker genes (*HAVCR2*, *CTLA4*, *LAG3*, *PDCD1*, and *BTLA*) were strongly correlated with GMFG expression (Table 3). These results indicate that GMFG might be a novel gene associated with immune cell infiltration and influencing microenvironment in gliomas.

**TABLE 3:** Correlation between GMFG expression and markers of immune cells in TCGA and CGGA datasets.

		TCGA				CGGA			
	Markers	Correlation	95% CI		*P*-value	Correlation	95% CI		*P*-value
CD8+ T-cell	CD8A	0.37	0.30	0.43	<0.0001	0.31	0.25	0.36	<0.0001
	CD8B	0.38	0.31	0.45	<0.0001	0.43	0.38	0.48	<0.0001
T-cell	CD3D	0.64	0.60	0.69	<0.0001	0.66	0.63	0.70	<0.0001
	CD3E	0.64	0.60	0.69	<0.0001	0.48	0.43	0.53	<0.0001
B cell	CD86	0.84	0.81	0.86	<0.0001	0.47	0.41	0.51	<0.0001
	CD79A	0.46	0.39	0.51	<0.0001	0.55	0.50	0.59	<0.0001
	CSF1R	0.64	0.59	0.68	<0.0001	0.29	0.23	0.35	<0.0001
Monocyte	CCL2	0.64	0.60	0.69	<0.0001	0.35	0.29	0.40	<0.0001
	CD68	0.86	0.83	0.88	<0.0001	0.43	0.38	0.48	<0.0001
M1 macrophage	CD86	0.83	0.81	0.86	<0.0001	0.47	0.41	0.51	<0.0001
	CD80	0.64	0.58	0.68	<0.0001	0.10	0.04	0.17	0.0008
	NOS2	0.02	−0.06	0.10	0.64	0.05	0.00	0.11	0.11
M2 macrophage	CD163	0.67	0.63	0.71	<0.0001	0.34	0.28	0.40	<0.0001
	MSR1	0.81	0.78	0.83	<0.0001	0.24	0.17	0.30	<0.0001
	MRC1	0.19	0.11	0.26	<0.0001	0.13	0.07	0.20	<0.0001
Neutrophil	ITGAM	0.71	0.67	0.74	<0.0001	0.43	0.37	0.48	<0.0001
	CCR7	0.52	0.46	0.57	<0.0001	0.18	0.12	0.24	<0.0001
	KIR2DL1	0.08	0.00	0.16	0.0405	–	–	–	
	KIR2DL3	0.17	0.10	0.25	<0.0001	–	–	–	
	KIR2DL4	0.43	0.36	0.49	<0.0001	–	–	–	
T-cell exhaustion	PDCD1	0.59	0.54	0.64	<0.0001	0.40	0.35	0.46	<0.0001
	CTLA4	0.53	0.47	0.58	<0.0001	0.34	0.28	0.39	<0.0001
	LAG3	0.38	0.31	0.44	<0.0001	0.57	0.53	0.61	<0.0001
	HAVCR2	0.86	0.84	0.88	<0.0001	0.37	0.31	0.42	<0.0001
	BTLA	0.46	0.39	0.52	<0.0001	0.21	0.15	0.27	<0.0001

To further validate the correlation between GMFG and immune infiltration in gliomas, we analyzed single-cell sequencing datasets of the gliomas from the TISCH database (Figure 5D). We found that the findings of the TISCH database were in line with the above results. GMFG expression was mainly associated with the infiltration of macrophages and monocytes (Figures 5E,F).

### Association Between Glia Maturation Factor-γ Expression and Tumor-Associated Macrophages Infiltration in Gliomas

**FIGURE 6:**
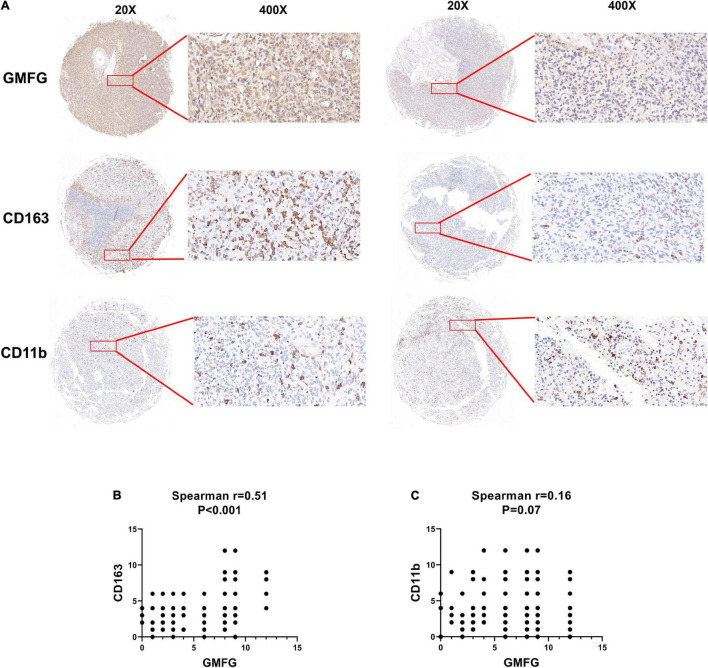
Association between GMFG expression and tumor-associated macrophages infiltration in gliomas. **(A)** We detected immune cells (CD11b^+^ and CD163^+^) infiltration in GBM and LGG samples using IHC staining, respectively. **(B,C)** Spearman correlation was employed to explore correlation between immune cells infiltration and GMFG expression. The IHC staining results were independently analyzed by two individuals.

Given the important role of macrophage infiltration in gliomas malignancy, the presence of different macrophage clusters is intriguing. Interaction between gliomas cells and the transformation of tumor-associated macrophages (TAMs) contribute to the rapid progression of gliomas (Chen et al., 2017). Therefore, we examined the infiltration level of immune cells (CD11b^+^ and CD163^+^) in gliomas samples by IHC staining. The results showed that high GMFG expression was strongly associated with higher infiltration of CD163^+^ macrophage cells (Figure 6A). In GBM, TAMs are the largest non-neoplastic cell type, constituting more than 30% of the tumor bulk and contributing significantly to tumor progression and treatment resistance (Hambardzumyan et al., 2016). Herein, the expression of GMFG was positively correlated with TAMs in gliomas (Figures 6B,C).

### High Glia Maturation Factor-γ Expression Predicted a Worse Prognosis in Gliomas

**FIGURE 7:**
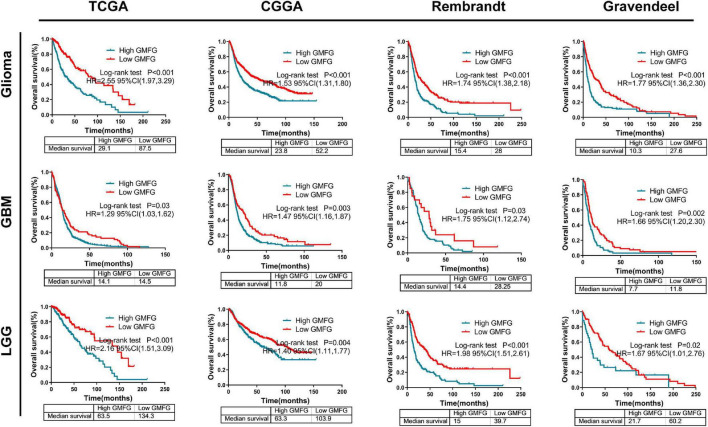
High GMFG expression predicted a worse prognosis in gliomas. Kaplan–Meier curves were used to plot overall survival curves against optimal cutoff in TCGA, CGGA, Gravendeel, and Rembrandt datasets. The optimal cutoff was determined using GlioVis. HR, hazard ratio.

Kaplan–Meier curves were used to plot the overall survival against optimal cutoff values. The optimal cutoff value was determined using GlioVis. The results showed that high GMFG expression in gliomas predicted a worse prognosis compared with low GMFG expression in TCGA, CGGA, Rembrandt, and Gravendeel datasets (Figure 7). Kaplan–Meier survival analyses were performed separately on the LGG and GBM. Interestingly, both GBM and LGG patients with high GMFG expression had shorter median survival than patients with low GMFG expression in all TCGA, CGGA, Gravendeel, and Rembrandt datasets (Figure 7). These results indicated that GMFG can predict the prognosis of patients with gliomas.

### Glia Maturation Factor-γ Expression Was Associated With Temozolomide Response to Gliomas

**FIGURE 8:**
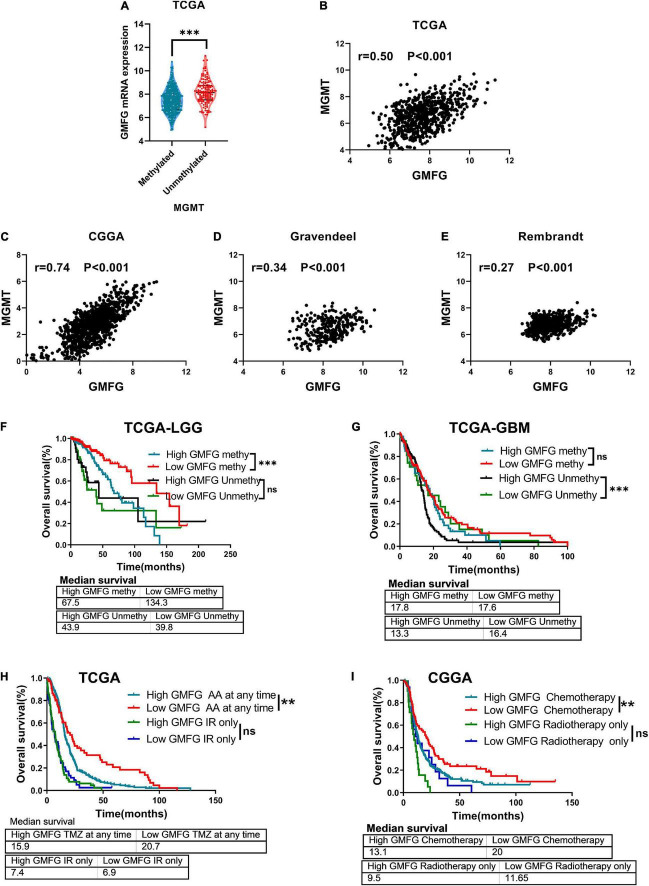
Glia maturation factor-γ associated with TMZ response of gliomas. **(A)** Association between GMFG expression and MGMT promoter methylation status. ^***^P < 0.001. **(B–E)** Correlation between GMFG expression and MGMT expression in TCGA, CGGA, Gravendeel, and Rembrandt datasets based on Spearman correlation analysis. **(F,G)** Effect of GMFG on the prognosis of patients with LGG and GBM with different MGMT promoter methylation status. HR, hazard ratio. ^***^P < 0.001, ns, no significance. **(H,I)** Effect of GMFG on the prognosis of gliomas patients who received chemotherapy at any time or radiotherapy only. AA, alkylating agent; IR, ion radiotherapy. **P < 0.01, ns, no significance.

The methylation of O6-methylguanine methyltransferase (MGMT) promoter inhibits the expression of MGMT, which increases the sensitivity of patients to TMZ treatment. In the TCGA dataset, we found that GMFG expression was significantly higher in gliomas with unmethylated MGMT promoter than in those with methylated MGMT promoter (Figure 8A). Moreover, GMFG expression was positively correlated with MGMT expression in all four public datasets (Figures 8B–E). These results indicated that GMFG may influence TMZ response in patients with glioma. Interestingly, we observed that GBM patients with high GMFG expression and unmethylated MGMT promoter had lower overall survival than patients with low GMFG expression and unmethylated MGMT promoter, but this was not the case for LGG patients (Figures 8F,G). GBM patients with high GMFG expression and unmethylated MGMT promoter had the worst prognosis among the four groups (median survival: 13.3 months) (Figure 8G). GMFG might serve as a supplement marker for treatment response predicting. Moreover, we found that patients with low GMFG expression who received chemotherapy had longer survival time than patients in the high GMFG group (MS: 20.9 vs. 15.9 months, 13.1 vs. 20.0 months, all *P* < 0.01, in CGGA and TCGA, respectively, Figures 8H,I). For patients who received ion radiotherapy (IR) only, GMFG expression had no effect on the overall survival neither in CGGA nor in TCGA datasets (Figures 8H,I).

## Discussion

Previously, the GMFG protein was not considered to participate in the development of gliomas (Peters et al., 1999). One study found that GMFG was markedly elevated in GBM, LGG, kidney clear carcinoma (KIRC), and acute myeloid leukemia (LAML) cancers (Lan et al., 2021). These findings were obtained only by means of bioinformatics analyses; thus, they should be validated in more glioma samples. In this study, we found that the GMFG was significantly upregulated in gliomas and its expression increased with glioma grade. Normalized RNA data from TCGA, CGGA, Rembrandt, and Gravendeel, as well as our in-house cohort containing 120 gliomas samples and 10 non-tumor brain tissues, were used for this study. High GMFG expression significantly correlated with the malignancy of gliomas and was strongly associated with IDH1/2 wild-type, 1p19q codeletion, and ME subtypes. Importantly, in the four public datasets, high GMFG expression predicted a worse prognosis of gliomas, indicating that GMFG can be a novel prognostic biomarker for patients with LGG and GBM.

Previous studies have revealed that GMFG mainly regulated filamentous actin structures and promoted migration and invasion of cancer cells (Ikeda et al., 2006; Zuo et al., 2014; Wang et al., 2017). GMFG can directly bind Arp2/3 complex and reorganize actin filaments, thereby enhancing cell migration (Yamaguchi et al., 2005). Epithelial to ME transition (EMT) is one of the main mechanisms driving the migration of glioma cells. Epithelial glioma cells lose their cell polarity, undergo cytoskeletal reorganization, and subsequently trans-differentiate into ME cells (Lamouille et al., 2014). In this study, GMFG was highly expressed in ME GBM and its expression significantly correlated with multiple ME-related genes. The previous study has designed to explore the impact of GMFG in pan-cancers and the results showed that GMFG was significantly upregulated in GBM (Lan et al., 2021). Our findings were consistent with our previous study, which provided more reliable confirmation of the role of GMFG in glioma. Indeed, IDH has been found to play an important role in the regulation of cell metabolism, which is a hallmark of epithelial to EMT in GBM (Lu et al., 2019). In addition, GMFG was significantly enriched in IDH wt gliomas, especially in GBM. Although we did not perform molecular biology confirmatory tests *in vitro*, we postulated that GMFG may regulate the EMT process.

Importantly, this study showed that GMFG is significantly associated with the infiltration of macrophages in the tumor microenvironment of gliomas. Results of GO enrichment analyses revealed that GMFG was primarily enriched processes that regulate innate/adaptive immune response or cytokine secretion. Furthermore, bioinformatic analyses indicated that GMFG may regulate remodeling of the immune environment or TAMs infiltration. In addition, the results of TISCH indicated that the main immune cells producing GMFG mRNA in glioma might be CD8 T cells and macrophages. Indeed, GMFG was previously reported to be an important regulator of T-cell and monocyte migration (Lippert and Wilkins, 2012; Aerbajinai et al., 2019). GMFG was also found to participate in the regulation of iron metabolism in macrophages and was responsible for the transition of macrophage phenotype (Lippert and Wilkins, 2012). Knock-down of GMFG in macrophages exhibited an iron deficiency response and enhanced expression of M2 macrophage markers toward the M2 phenotype (Sarkar et al., 2014). These findings demonstrate the potential role of GMFG in the regulation of infiltration of TAMs in gliomas. Indeed, correlations between GMFG and immune cell infiltration were also analyzed by Lan et al. (2021), while we provided more details. Positive correlations between GMFG expression and immune cell infiltration had been validated by using the public databases ESTIMATE, TIMER, and the single-cell database (TISCH). To further validate our findings, we performed IHC staining for one of the TAMs markers (CD163) and found that GMFG expression was significantly associated with CD163 expression in gliomas. The infiltration of TAMs in gliomas contributes to the rapid progression of glioma malignancy (Stepanenko and Chekhonin, 2019). In our study, IHC stainings and bioinformatic analyses further indicated that GMFG expression correlated with the infiltration of TAMs.

The TMZ is the first-line therapy for gliomas. The methylation status of the MGMT promoter is the only marker used to evaluate TMZ treatment response. So far, the use of MGMT as a marker of TMZ response is highly controversial because of its questionable accuracy (Stepanenko and Chekhonin, 2019; Herbener et al., 2020). In this study, we demonstrate that GMFG can be a complementary marker when combined with the methylated status of MGMT promoter for predicting TMZ response in gliomas. Studies have shown that immune cell infiltration in the gliomas microenvironment can affect glioma cell proliferation, invasion, and chemotherapy resistance (Gregoire et al., 2020). In this study, glioma patients with higher immune cell infiltration had a survival shorter time compared with those with lower immune cell infiltration. Our analyses regarding GMFG expression and cell markers were consistent with GMFG association with the remodeling of the tumor microenvironment and TAM infiltration in gliomas. Indeed, the impact of TAMs and TMZ treatment were found to affect each other. CD74 in TAMs was reported to enhance the TMZ resistance by activating AKT and Erk1/2 pathways (Kitange et al., 2010). A previous study has revealed that increased CD163+ macrophages not only enhanced cancer stemness but also correlated with TMZ resistance in gliomas (Kazantseva et al., 2018). Therefore, we speculate that GMFG regulates the infiltration of M2 macrophages, which in turn promotes TMZ resistance in glioma cells. This study presents a novel gene that might determine the relationship between TAM and TMZ response in gliomas.

## Conclusion

Glia maturation factor-γ is a novel gene that is strongly correlated with the malignancy of gliomas. It can also be used as a prognostic biomarker in patients with both LGG and GBM. Increased GMFG expression is associated with TAM infiltration and a bad response to TMZ treatment.

## Data Availability Statement

The datasets presented in this study can be found in online repositories. The names of the repository/repositories and accession number(s) can be found below: http://gliovis.bioinfo.cnio.es/, http://gdac.broadinstitute.org/, http://www.cgga.org.cn/, and https://www.cbioportal.org/.

## Ethics Statement

Approval for this study was issued by the Institutional Ethics Committee of the Faculty of Medicine at Renmin Hospital of Wuhan University [approval number: 2012LKSZ (010) H]. The patients/participants provided their written informed consent to participate in this study.

## Author Contributions

JL, XZ, and ZC contributed to the conception of the study. LG and RG performed the IHC experiment. XT contributed significantly to the analysis and manuscript preparation. JL, XZ, and XT performed the data analyses. JL wrote the manuscript. ZC and HX were responsible for proofreading. All authors contributed to the article and approved the submitted version.

## Conflict of Interest

The authors declare that the research was conducted in the absence of any commercial or financial relationships that could be construed as a potential conflict of interest.

## Publisher’s Note

All claims expressed in this article are solely those of the authors and do not necessarily represent those of their affiliated organizations, or those of the publisher, the editors and the reviewers. Any product that may be evaluated in this article, or claim that may be made by its manufacturer, is not guaranteed or endorsed by the publisher.

## Footnotes

1
www.oncomine.org

2
http://www.cbioportal.org/

3
http://gliovis.bioinfo.cnio.es/

4
http://metascape.org/

5
https://cistrome.shinyapps.io/timer
