# Supplementary figures and images for: Expression and Prognostic Role of Glia Maturation Factor-γ in Gliomas
Source: Front Mol Neurosci. 2022 Jun 29;15:906762. doi: 10.3389/fnmol.2022.906762 (PMC9277395; doi:10.3389/fnmol.2022.906762)

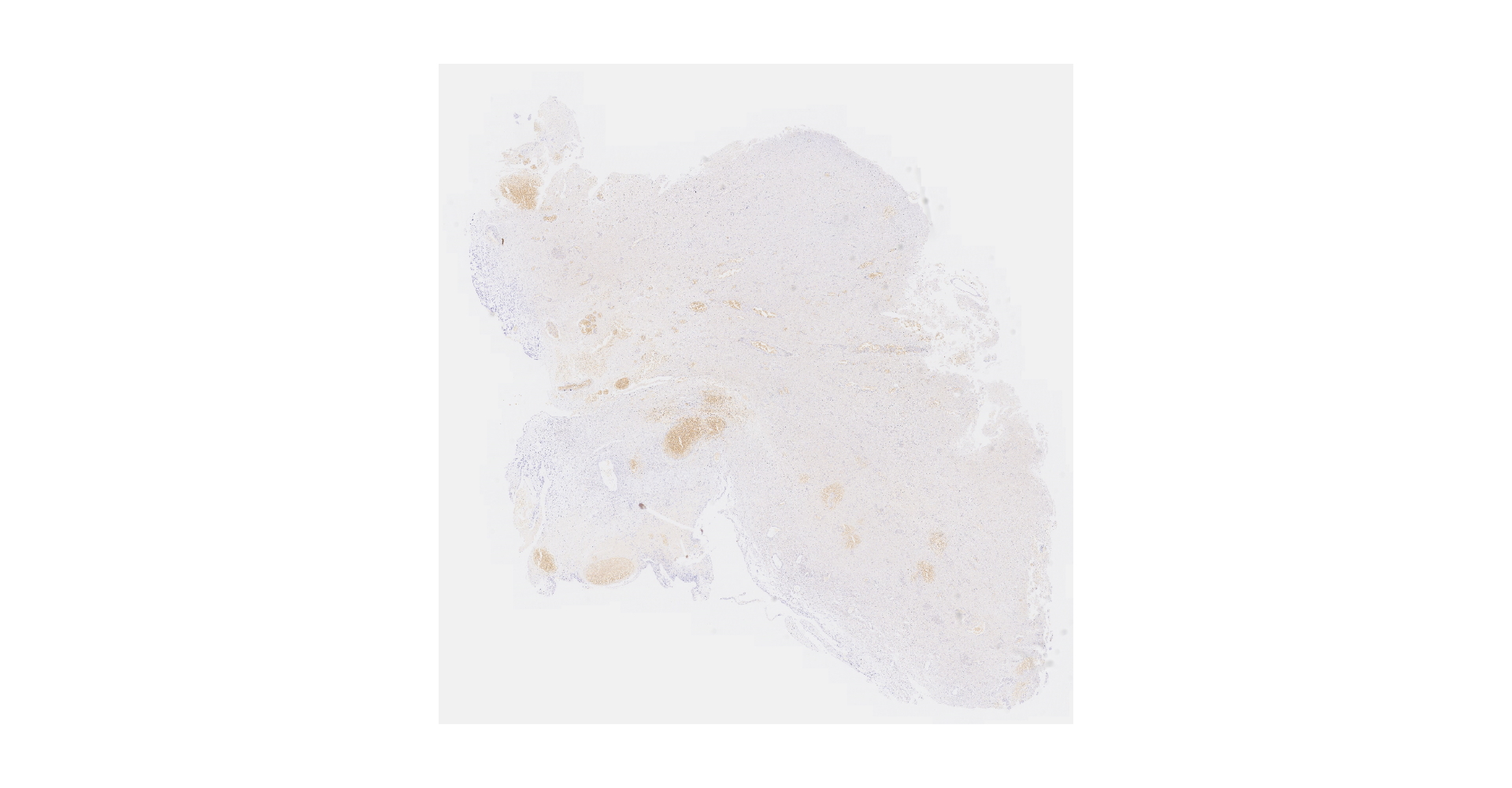

Supplement: Supplementary file 1 [file Image_1.JPEG]

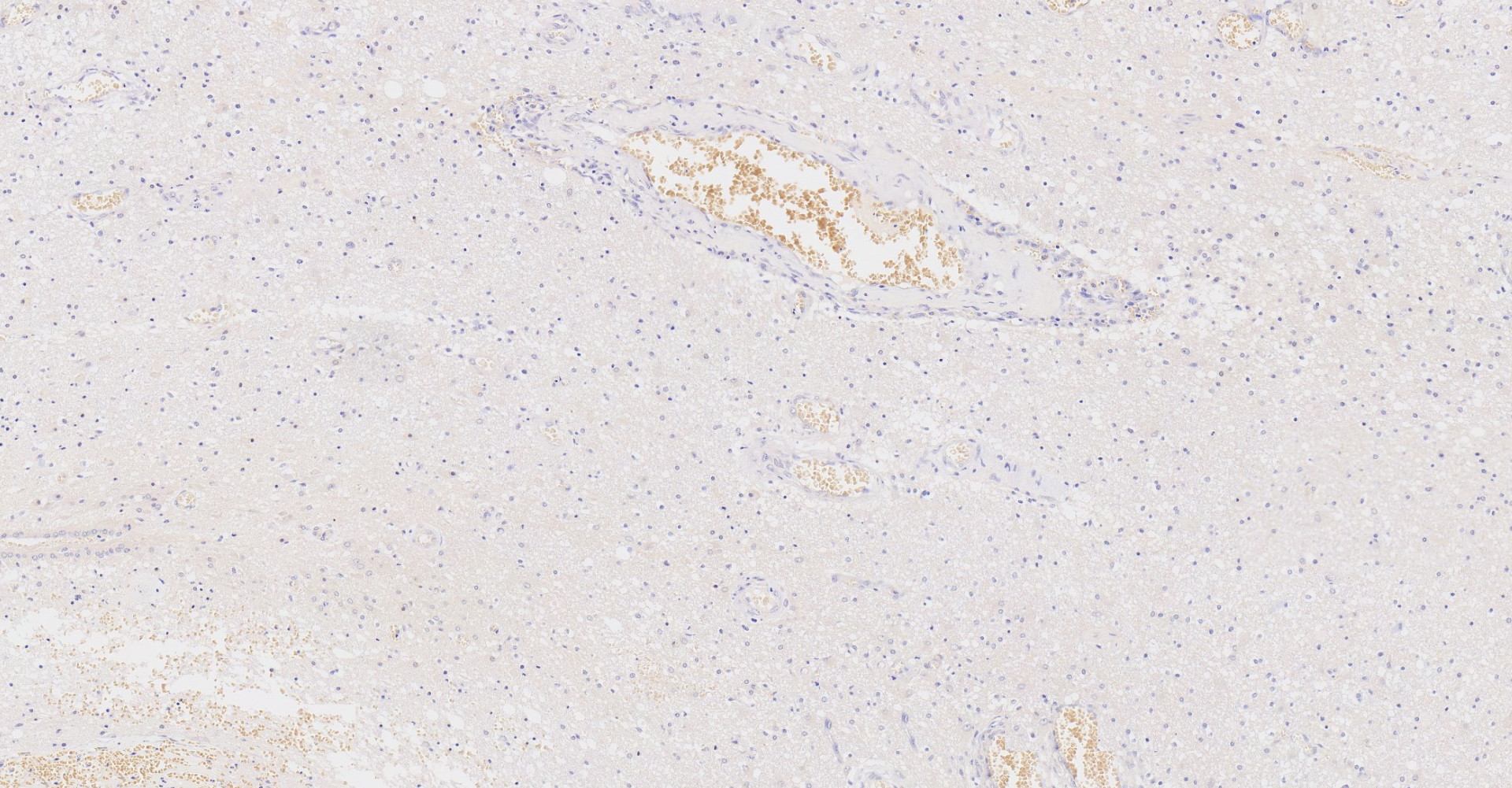

Supplement: Supplementary file 2 [file Image_2.JPEG]

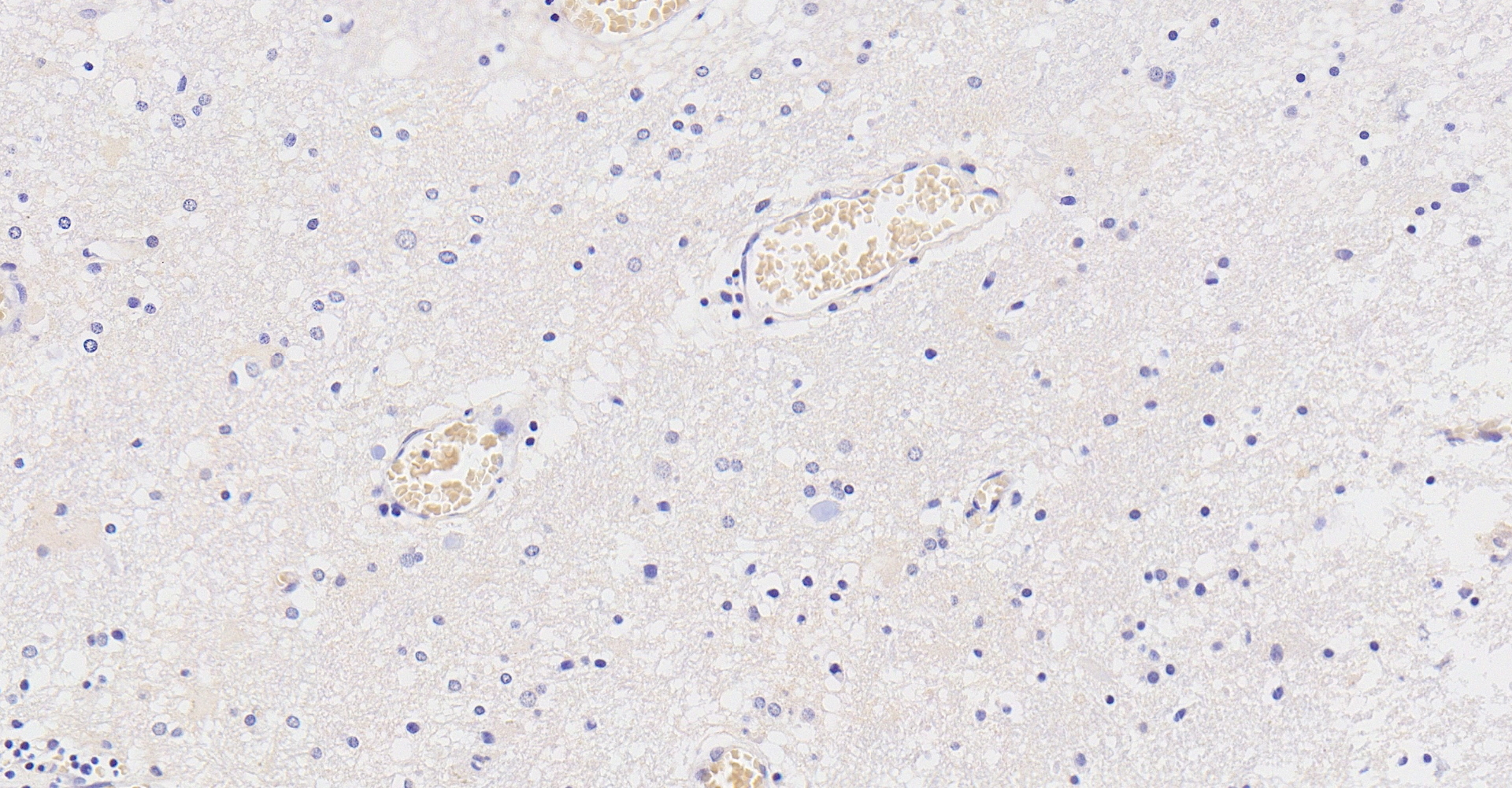

Supplement: Supplementary file 3 [file Image_3.JPEG]

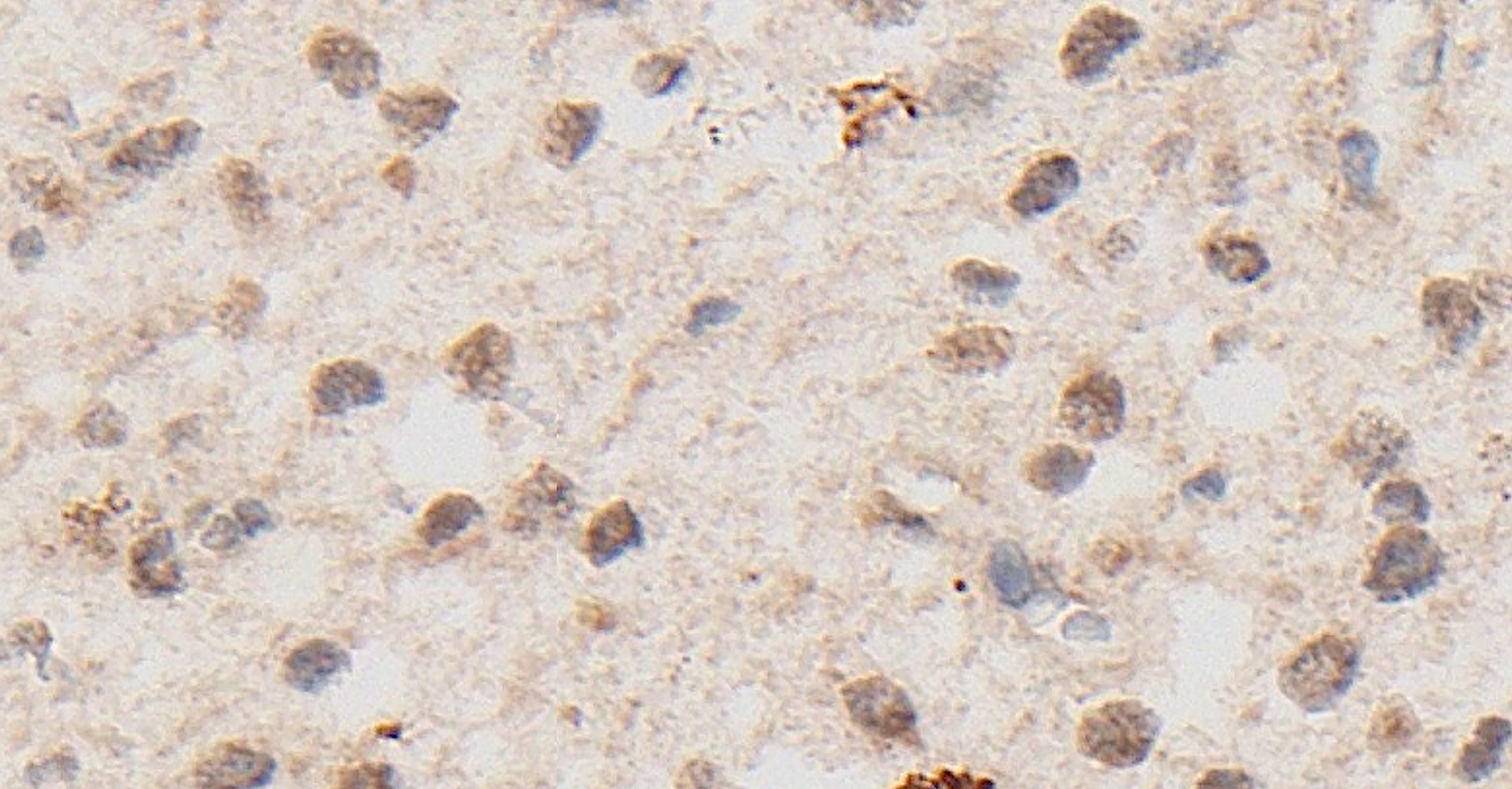

Supplement: Supplementary file 4 [file Image_4.TIF]

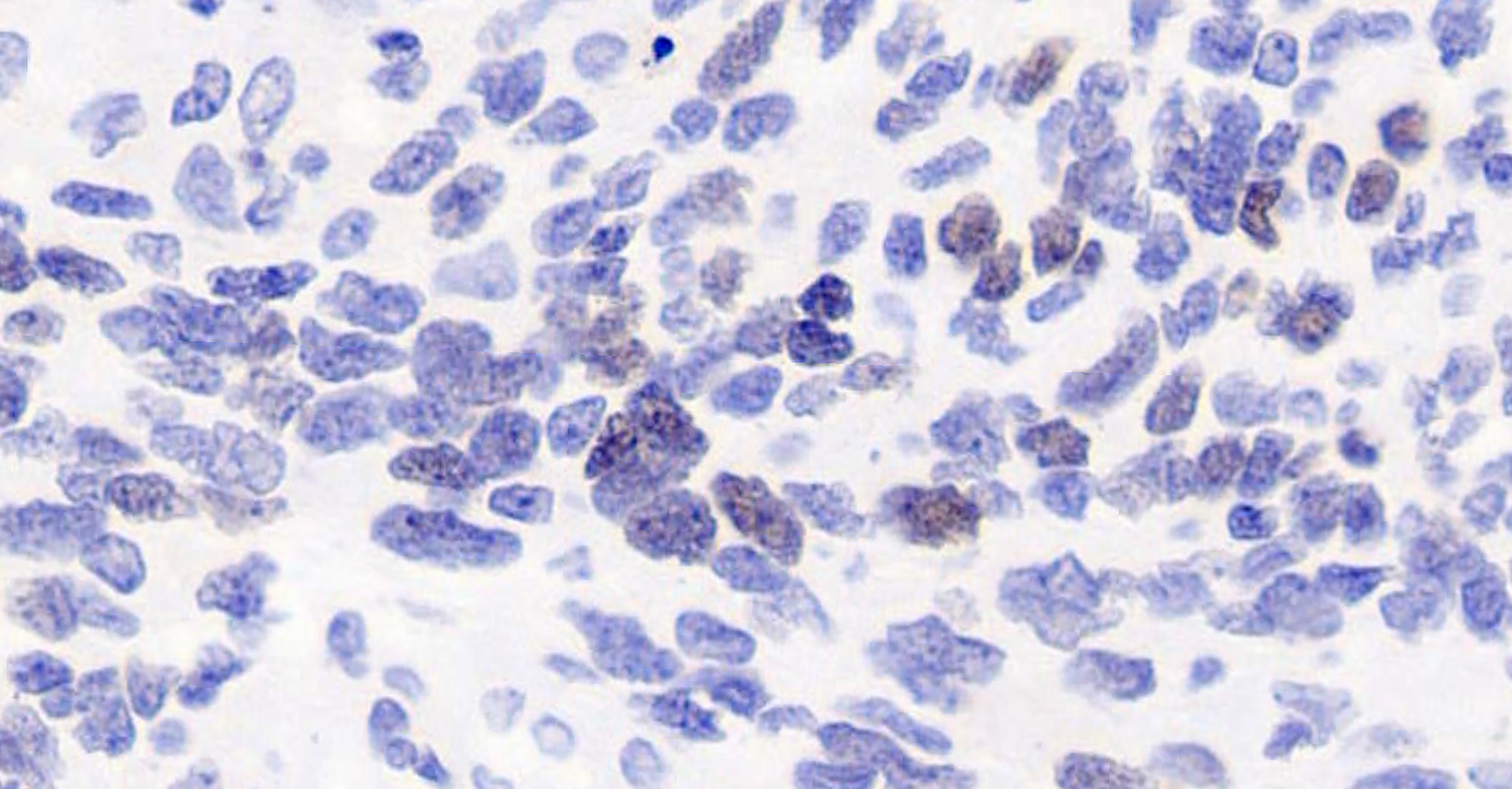

Supplement: Supplementary file 5 [file Image_5.TIF]

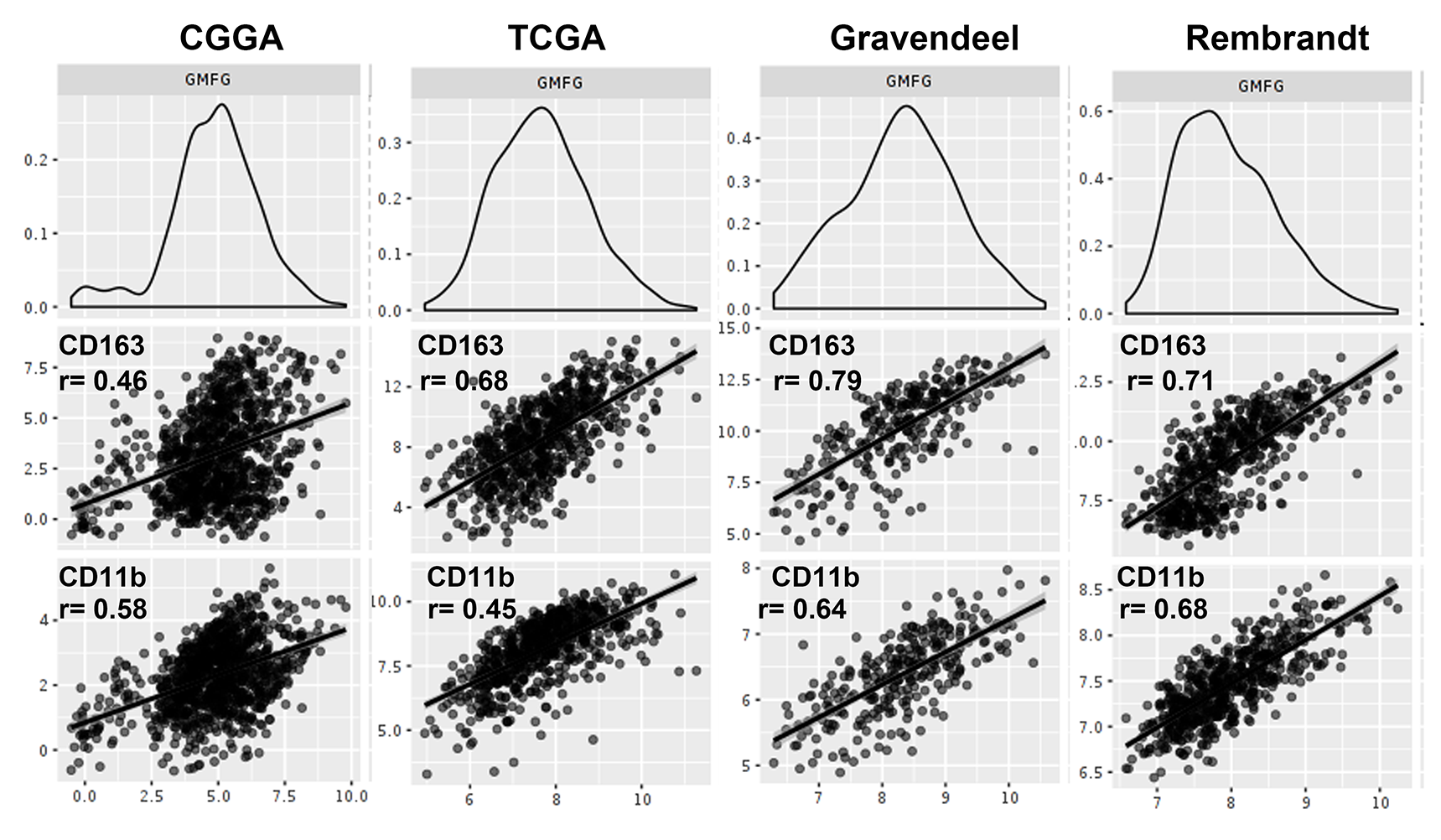

Supplement: Supplementary file 6 [file Image_6.TIF]
