# Supplementary material for: Expression and Prognostic Role of Glia Maturation Factor-γ in Gliomas
Source: Front Mol Neurosci. 2022 Jun 29;15:906762. doi: 10.3389/fnmol.2022.906762 (PMC9277395; doi:10.3389/fnmol.2022.906762)
Supplement: Supplementary file 7 [file Table_1.docx]

**Supplementary Table 1**

Marker genes collected and curated for major-lineage level of cell-type annotation.

| **Major-lineage cell type** | **Markers** |
| --- | --- |
| B | ABCB4, ADAM28, BACH2, BANK1, BCL7A, BEND5, BLK, BRAF, CD180, CD19, CD1C, CD22, CD37, CD69, CD72, CD79A, CD79B, CR2, CXCR5, EAF2, FAIM3, FCER2, FCGR2B, FCRL2, FRK, GPR18, GUSBP11, HHEX, HLA-DOB, IGHD, IGHM, IGKC, IGLL3P, IL4R, IRF8, KIAA0226L, LINC00921, LTB, LY86, MEP1A, MICAL3, MS4A1, NIPSNAP3B, NMBR, P2RX5, P2RY14, PNOC, PSG2, PTPRCAP, RALGPS2, RASGRP2, SELL, SIK1, SLC12A1, SPIB, STAP1, TCL1A, UGT1A8, VPREB3, ZNF286A, AIM2, ALOX5, CCR6, CD27, CLCA3P, DENND5B, FAM65B, GNG7, IFNA10, IL7, MBL2, NPIPB15, SIT1, SP140, TMEM156, TNFRSF13B, TNFRSF17, TRAF4, ZBTB32 |
| CD4Tconv | ACAP1, ANKRD55, ATHL1, BCL11B, CCR7, CD2, CD247, CD27, CD3D, CD3G, CD40LG, CD7, CXorf57, DPP4, DSC1, EPHA1, FAIM3, FLJ13197, FLT3LG, GAL3ST4, GALR1, GPR1, GRAP2, GZMM, ICOS, IL7R, ITK, LAT, LCK, LEF1, LIME1, LTB, LY9, MAP4K1, MAP4K2, MAP9, RASGRP2, RPL3P7, SERGEF, SH2D1A, SIRPG, TCF7, TRAC, TRAT1, TRAV13-1, TRBC1, UBASH3A, VILL, WNT7A, ZAP70, ZNF204P, ZNF324, CCL5, CCR6, CD28, CD3E, CD4, CD6, CD69, CD96, CTLA4, CTSW, DGKA, EPB41, ETS1, FBXL8, GPR171, GPR25, GZMA, GZMK, KLRB1, NKG7, PBXIP1, PTGER2, PTPRCAP, RASA3, RCAN3, RPL10L, ST8SIA1, TRAV13-2, TRAV21, TRAV8-6, TRAV9-2, ZFP36L2, CCL20, CDC25A, CSF2, CXCL13, GPR19, GZMB, IFNG, IL12RB2, IL17A, IL26, IL2RA, IL3, IL4, IL9, LAG3, LTA, ORC1, PMCH, RRP9, SKA1, TNFRSF4, TNIP3, CHI3L2, CXCR5, FOSB, FZD3, ICA1, IL21, PASK, PDCD1, PVRIG, RGS1, SIK1, SLC7A10, TRIB2, TSHR, ZBTB10 |
| CD8T | BCL11B, CCL5, CD2, CD247, CD27, CD3D, CD3E, CD3G, CD6, CD69, CD7, CD8A, CD8B, CD96, CRTAM, CST7, CTSW, DPP4, DSC1, DUSP2, FAIM3, FLT3LG, GNLY, GPR171, GRAP2, GZMA, GZMB, GZMH, GZMK, GZMM, ICOS, IGKC, IL7R, ITK, KLRB1, KLRC3, KLRC4, KLRD1, KLRF1, KLRK1, LAG3, LCK, LEF1, LIME1, LTB, LY9, MAP4K1, MAP9, NCR3, NKG7, PIK3IP1, PRF1, PTGDR, PTPRCAP, PVRIG, RASA3, RPL3P7, SH2D1A, SIRPG, TCF7, TRAC, TRAT1, TRAV12-2, TRAV13-1, TRBC1, TRDC, UBASH3A, ZAP70 |
| CD8Tex | BCL11B, CCL5, CD2, CD247, CD27, CD3D, CD3E, CD3G, CD6, CD69, CD7, CD8A, CD8B, CD96, CRTAM, CST7, CTSW, DPP4, DSC1, DUSP2, FAIM3, FLT3LG, GNLY, GPR171, GRAP2, GZMA, GZMB, GZMH, GZMK, GZMM, ICOS, IGKC, IL7R, ITK, KLRB1, KLRC3, KLRC4, KLRD1, KLRF1, KLRK1, LAG3, LCK, LEF1, LIME1, LTB, LY9, MAP4K1, MAP9, NCR3, NKG7, PIK3IP1, PRF1, PTGDR, PTPRCAP, PVRIG, RASA3, RPL3P7, SH2D1A, SIRPG, TCF7, TRAC, TRAT1, TRAV12-2, TRAV13-1, TRBC1, TRDC, UBASH3A, ZAP70, PDCD1, CTLA4, TIGIT, HAVCR2 |
| DC | AIF1, ALOX15, C1orf54, CCDC102B, CCL13, CCL17, CCL18, CCL22, CD1A, CD1B, CD1C, CD1E, CD209, CD33, CD68, CLEC10A, CLEC4A, CLEC7A, CLIC2, DHRS11, EGR2, FAM198B, FCER1A, FCER2, FLVCR2, FPR3, FZD2, HLA-DQA1, IGSF6, MMP12, NCF2, PLA2G7, PPFIBP1, RNASE6, SCN9A, SLAMF8, SLC15A3, TMEM255A, TREM2, ARHGAP22, BIRC3, CCL1, CCL19, CCL20, CCL5, CCL8, CCR7, CD80, CD86, CHST7, CXCL10, CXCL11, CYP27A1, DHX58, EBI3, ETV3, HESX1, HTR2B, IDO1, IFI44L, IL12B, IL2RA, KYNU, LAMP3, MAP3K13, MSC, NR4A3, PDCD1LG2, PLA1A, PTGIR, RASSF4, RSAD2, SIGLEC1, SLC2A6, SLCO5A1, ST3GAL6, TNFAIP6, TNFRSF11A, TNFRSF4 |
| Endothelial | PECAM1, VWF, ENG |
| Fibroblasts | FAP, PDPN, MMP2, PDGFRA, THY1, MMP11, PDGFRL, TGFB3, COL1A2, DCN, COL3A1, COL6A1 |
| Mast | ATP8B4, BMP2K, BPI, C3AR1, CD33, CEACAM8, CLC, CMA1, CPA3, CRISP3, CTSG, FAM124B, FAM174B, FCER1A, GFI1, HDC, HPGDS, IL18R1, LTC4S, MS4A2, MS4A3, MYB, NOX3, NTRK1, P2RX1, P2RY14, PAQR5, PRG2, RAB27B, RGS13, SEPT8, SLC12A8, ST8SIA1, STAP1, STXBP6, TPSAB1, CCL1, CCL20, CCL4, CSF2, CXCL3, GZMB, HOXA1, IL1A, IL1B, IL1RL1, IL3, IL5, LINC00597, MARCH3, TEC |
| Mono/Macro | AIF1, APOBEC3A, AQP9, ASGR1, ASGR2, BST1, C5AR1, CCR2, CD1D, CD33, CD68, CDA, CFP, CHST15, CLEC4A, CLEC7A, CREB5, CSF3R, FAM198B, FCN1, FES, FOSB, FPR1, FZD2, HCK, HK3, HNMT, HPSE, IGSF6, LILRA2, LILRA3, LILRB2, LST1, MEFV, MNDA, MS4A6A, NCF2, NFE2, NLRP3, NOD2, P2RY13, PADI4, RNASE2, RNASE6, S100A12, SLC15A3, TLR2, TLR7, TLR8, UPK3A, VNN1, VNN2, ACP5, ADAMDEC1, BHLHE41, CCDC102B, CCL18, CCL22, CCL7, CHI3L1, COL8A2, CSF1, CXCL3, CXCL5, CYP27A1, DCSTAMP, GPC4, MARCO, MMP9, PLA2G7, PPBP, QPCT, SLAMF8, SLC12A8, TNFSF14, TREM2, ACHE, APOL3, APOL6, ARRB1, CCL19, CCL5, CCL8, CCR7, CD38, CD40, CLIC2, CXCL10, CXCL11, CXCL13, CXCL9, CYP27B1, DHX58, EBI3, GGT5, HESX1, IDO1, IFI44L, IL2RA, KIAA0754, KYNU, LAG3, LAMP3, PLA1A, PTGIR, RASSF4, RSAD2, SIGLEC1, SLAMF1, SLC2A6, SOCS1, TNFAIP6, TNIP3, TRPM4, ALOX15, CCL13, CCL14, CCL23, CD209, CD4, CLEC10A, CRYBB1, FRMD4A, GSTT1, HRH1, HTR2B, NME8, NPL, PDCD1LG2, RENBP, WNT5B |
| Myofibroblasts | ACTA2, MCAM, MYLK, MYL9, IL6, PDGFA |
| Neutrophils | AIF1, APOBEC3A, AQP9, BTNL8, C5AR1, CAMP, CASP5, CCR3, CDA, CEACAM3, CFP, CHI3L1, CHST15, CLC, CREB5, CSF3R, CXCR1, CXCR2, DPEP2, EMR2, EMR3, FAM212B, FCGR3B, FFAR2, FPR1, FPR2, GPR97, HAL, HSPA6, IGSF6, IL18RAP, LILRA2, LILRB2, LST1, MAK, MEFV, MGAM, MMP25, MNDA, MXD1, NCF2, NFE2, P2RY13, P2RY14, PADI4, PGLYRP1, PLEKHG3, QPCT, REPS2, S100A12, STEAP4, TLR2, TLR8, TNFAIP6, TNFRSF10C, TREM1, TREML2, VNN1, VNN2, VNN3 |
| NK | BPI, CAMP, CCL5, CD160, CD2, CD244, CD247, CD7, CD96, CDHR1, CEACAM8, CST7, CTSW, DEFA4, ELANE, GFI1, GNLY, GZMA, GZMB, GZMH, GZMK, GZMM, IL12RB2, IL18R1, IL18RAP, IL2RB, KIR2DL1, KIR3DL2, KLRB1, KLRC3, KLRC4, KLRD1, KLRF1, KLRK1, LCK, MGAM, MS4A3, NAALADL1, NKG7, NME8, PLEKHF1, PRF1, PRR5L, PTGDR, PTPRCAP, PVRIG, S1PR5, SH2D1A, TBX21, TEP1, TRBC1, TRDC, TTC38, TXK, ZAP70, ZNF135, APOBEC3G, APOL6, CCL4, CCND2, CD69, CDK6, CSF2, DPP4, FASLG, GPR171, GPR18, GRAP2, IFNG, KIR2DL4, KIR2DS4, LTA, LTB, NCR3, OSM, PTGER2, SOCS1, TNFSF14 |
| pDC | AIF1, ALOX15, C1orf54, CCpDC102B, CCL13, CCL17, CCL18, CD1A, CD1B, CD1C, CD1E, CD33, CD68, CLEC10A, CLEC4A, CLEC7A, CLIC2, DHRS11, EGR2, FAM198B, FCER1A, FCER2, FLVCR2, FPR3, FZD2, HLA-DQA1, IGSF6, MMP12, NCF2, PLA2G7, PPFIBP1, RNASE6, SCN9A, SLAMF8, SLC15A3, TMEM255A, TREM2, CLEC4C, IRF7, LILRB4, Siglech, ARHGAP22, BIRC3, CCL1, CCL19, CCL20, CCL5, CCL8, CCR7, CD80, CD86, CHST7, CXCL10, CXCL11, CYP27A1, DHX58, EBI3, ETV3, HESX1, HTR2B, IDO1, IFI44L, IL12B, IL2RA, KYNU, LAMP3, MAP3K13, MSC, NR4A3, PpDCD1LG2, PLA1A, PTGIR, RASSF4, RSAD2, SIGLEC1, SLC2A6, SLCO5A1, ST3GAL6, TNFAIP6, TNFRSF11A, TNFRSF4 |
| Plasma | ABCB9, AMPD1, ANGPT4, ATXN8OS, C11orf80, CCR10, CD27, CD38, CD79A, DENND5B, EAF2, FCRL2, GNG7, GPR25, GUSBP11, HIST1H2AE, HIST1H2BG, HLA-DOB, IGHD, IGHE, IGHM, IGKC, IGLL3P, KCNA3, KCNG2, LIME1, LOC100130100, MAN1A1, MANEA, MAST1, MROH7, MZB1, P2RX5, PAX7, PDK1, PNOC, RASGRP3, REN, RGS13, RPL3P7, SIK1, SPAG4, ST6GALNAC4, TGM5, TMEM156, TNFRSF17, UGT2B17, ZBP1, ZNF165 |
| TMKI67 | BCL11B, CD2, CD247, CD27, CD28, CD3D, CD3E, CD3G, CD6, CD7, CD8A, CD8B, CD96, CXCR6, FLT3LG, FYN, GIMAP4, GPR171, GZMK, GZMM, ICOS, ITK, LCK, LIME1, PRKCH, PSTPIP1, SH2D1A, SIRPG, TNFRSF9, TRAC, TRAT1, TRBC1, TRBC2, UBASH3A, ZAP70, AURKA, BIRC5, BUB1, CCNA2, CCNB1, CDC20, CDK1, CDKN3, FEN1, HMGB2, MCM2, MCM5, MCM6, MYBL2, NUSAP1, PCNA, PLK1, TOP2A, ZWINT |
| Treg | BARX2, BCL11B, CD2, CD247, CD27, CD28, CD3D, CD3E, CD3G, CD4, CD5, CD6, CD70, CD96, CEMP1, CLEC2D, CTLA4, DGKA, DPP4, EFNA5, FOXP3, FRMD8, GPR1, GPR171, GPR19, GZMM, HIC1, HMGB3P30, ICOS, IL2RA, IL2RB, ITK, KIRREL, LAIR2, LCK, LILRA4, LOC126987, LTB, MAP4K1, MBL2, NPAS1, NTN3, PCDHA5, PLCH2, PMCH, PTGIR, PTPRG, RCAN3, RYR1, SEC31B, SEPT5, SH2D1A, SIRPG, SIT1, SKAP1, SPOCK2, SSX1, TRAC, TRAT1, TRAV9-2, TRBC1, TYR, UBASH3A, ZAP70 |
